# Children's oral prevention: a continuum from pregnancy: Comment to: Impact of dental orientation given to mothers during pregnancy on oral health of their children. Einstein (Sao Paulo). 2016;14(2):219-25

**DOI:** 10.1590/S1679-45082016CE3804

**Published:** 2016

**Authors:** Marcos Roberto Tovani-Palone

**Affiliations:** Hospital de Reabilitação de Anomalias Craniofaciais, Universidade de São Paulo, Bauru, Brazil

Dear Editor,

The findings of the study by Rigo et al.^(1)^ are of great importance concerning prevention in children's oral health. However, we should highlight that extend this type of approach for babies by dental clinics is quite relevant. An example is the Hospital for Rehabilitation of Craniofacial Anomalies (Bauru, (SP), Brazil), which provide information on proper tooth brushing and adequate use of toothpaste.^(2)^ In addition, there is an emphasis on issues related to dental caries and periodontal diseases as barriers for surgeries,^(3)^ and also risky food habits^(4)^ and possible dental changes in deciduous and permanent teeth.^(5)^ Programs to promote early awareness of pregnant women on children's oral care, as well as dental care for babies are important preventive measures to wider Public Health Policies, especially in outpatient and hospital services focused on this public throughout the country.
